# Evaluating binaural hearing capabilities in individuals with sensorineural hearing loss through bilateral bone conduction stimulation

**DOI:** 10.1038/s41598-024-80379-1

**Published:** 2024-11-21

**Authors:** Stefan Stenfelt, Mehrnaz Zeitooni, Elina Mäki-Torkko

**Affiliations:** 1 Department of Biomedical and Clinical Sciences, Linköping University, Linköping, 58185 Sweden; 2 School of Medical Sciences, Örebro University, Örebro, 701 82 Sweden

**Keywords:** Biomedical engineering, Auditory system

## Abstract

This study investigated the impact of bilateral bone conduction (BC) stimulation and sensorineural hearing loss on spatial release from masking, binaural intelligibility level difference, and lateralization. The study involved two groups of adults with mild to moderate sensorineural hearing loss: one group of 21 participants with symmetric hearing loss and another group of nine participants with asymmetric hearing loss. All tests were conducted through BC and air conduction (AC) headsets with non-individualized virtual positions of the sound sources and linear amplification based on individual hearing thresholds. The findings revealed a bilateral benefit for both groups of hearing-impaired individuals, with symmetric hearing loss yielding better results than asymmetric hearing loss. AC stimulation provided approximately twice the benefit in terms of dB compared to BC stimulation. A large part of this benefit originated from a favorable signal-to-noise ratio due to noise reduction from the head shadow. However, binaural processing was present in both hearing-impaired groups with bilateral BC stimulation. The ability to lateralize sounds based on interaural time delays was significantly impaired in participants with both types of hearing loss when stimulation was by BC. Despite these challenges, the study underscores the benefits of bilateral fitting of BC hearing aids, even in individuals with mild to moderate sensorineural hearing loss, whether symmetric or asymmetric.

## Introduction

Binaural hearing, or binaural processing, refers to the brain’s ability to integrate auditory information from both ears to improve speech understanding in challenging environments and localize sound sources^1–3^. This process utilizes the similarities and differences in sounds received by each ear to gather information about the surrounding acoustic environment. The two primary cues for binaural processing are the differences in time of arrival (interaural time difference, ITD) or phase (interaural phase difference, IPD) for stationary sounds, and the difference in sound level (interaural level difference, ILD)^4^. By using these cues, the auditory system can accurately determine the direction and distance of a target sound source, thereby improving its detection^5^.

In contrast, bilateral hearing refers to the detection of sound by both ears, regardless of whether the auditory information is process jointly by the brain^6^. Although both ears may detect sound in bilateral hearing, there may be minimal or no integration of auditory information between them. Thus, bilateral hearing involves the simple detection of sound with both ears, whereas binaural hearing enables sound source localization and noise suppression through integrated processing.

Research consistently shows that individuals with sensorineural hearing loss, as measured by audiograms (pure-tone detection), generally exhibit reduced binaural abilities compared to those with normal hearing^7–10^. These deficits manifest in difficulties with sound localization and poorer speech perception in complex listening environments^10,11^. While factors such as age and cognitive function can also influence binaural processing, the integrity of the peripheral auditory system plays a key role in ensuring optimal binaural function^12^.

Asymmetric sensorineural hearing loss has a greater impact on binaural abilities than symmetric hearing loss^13^. Asymmetric hearing loss can distort binaural cues, leading to significant disruptions in binaural hearing^7,14^. Although individuals with asymmetric hearing loss may have similar speech understanding in noise as individuals with symmetric hearing loss when both speech and noise is collocated in front of them^15^, their ability to benefit from spatial separation of speech and noise is reduced compared to those with symmetric hearing loss^16,17^. Additionally, sound source localization is significantly worse in individuals with asymmetric hearing loss compared to those with symmetric hearing loss^14,18^.

When a sound is delivered in a sound field or via traditional earphones, the primary transmission pathway is air conduction (AC). This means that the sound travels through the ear canal and middle ear ossicles^19^. Another mode of hearing sound is through bone conduction (BC), where the sound reaches the inner ear via vibrations in the skull bone and soft tissues^20–22^. Although BC sound has pathways through the ear canal and via the middle ear similar to AC sound transmission, the primary stimulation for BC sound applied at the mastoid in a healthy ear is through vibration of the skull bone^23–26^.

Binaural processing, which enhances hearing, is also observed when sound is delivered bilaterally by BC^27,28^. This has been demonstrated through spatial benefits in speech-in-noise tests, where bilateral BC stimulation improved performance, both in experimental^27–29^ and clinical settings^30–32^. Additionally, the ability to localize sound sources further supports the presence of binaural processing with bilateral BC sound^33–35^. However, it is important to note that while these studies have shown binaural benefits with BC, the advantages are typically less pronounced compared to bilateral sound delivered through AC^28,29,36^.

One argument against bilateral BC stimulation is that the sound from one stimulation position reaches both ears^37–40^. This suggests that it would be sufficient to stimulate both cochleae from one position, and that binaural cues are impeded due to cross hearing^36,40,41^. A recent study on bilateral BC stimulation found that the average low-frequency interaural time delay (at 500 Hz) was 0.2 ms and the level difference was close to 10 dB^42^. Even though this suggests that cross-hearing distorts binaural cues, these differences should still allow for the use of binaural information. This was confirmed in studies where subjects with normal hearing were stimulated bilaterally by BC, and speech perception in noise improved when speech and noise were spatially separated^27,28^. However, these studies also showed that the improvement was less with BC stimulation compared to stimulation by earphones (AC).

Previous studies have shown that using BC hearing aids on both ears can improve speech comprehension and sound localization, compared to using a BC hearing aid on only one ear^31,35,43,44^. However, these studies often involve small, heterogenous groups of patients with varying degrees of sensorineural hearing loss^31,32,35^. As a result, the combined impact of sensorineural hearing loss and cross-head transmission, present with BC stimulation, on the binaural benefits of hearing through bilateral BC devices is not fully understood.

Both sensorineural hearing loss and cross-head sound transmission can interfere with binaural hearing benefits. Therefore, it remains unclear at what level of hearing loss the advantages of binaural processing become negligible when sound is delivered bilaterally via BC. Furthermore, asymmetric hearing loss presents an even greater challenge to binaural integration compared to symmetric hearing loss, but the extent of asymmetry that still allows for meaningful binaural benefit, considering cross-head transmission, is currently unknown.

Understanding this relationship is critical for determining candidacy for bilateral BC hearing aid use. The aim of this study is to investigate how varying degrees of symmetric and asymmetric sensorineural hearing loss affect binaural benefits when sound is delivered bilaterally via BC devices. This knowledge will help optimize the application of bilateral BC hearing aids and improve patient outcomes.

In this study, binaural processing was evaluated using three different methods. The first method, spatial release from masking (SRM), assessed the benefit of spatially separating speech and noise sources^45,46^. SRM is influenced by two mechanisms: the better-ear effect and binaural unmasking. The better-ear effect refers to the improvement in the signal-to-noise ratio (SNR) at one ear due to the relative positioning of speech and noise sources. To isolate the better-ear effect, the SRM was measured under both binaural and monaural conditions, with monaural stimulation at the ear with the more favorable SNR.

To evaluate the binaural unmasking in speech-in-noise, binaural intelligibility level difference (BILD) was measured^47^. In this test, the speech signal was either presented equally or phase-inverted between the two ears, while the noise remained identical at both ears. Since the SNR at both ears was the same, any improvement in speech perception was attributed solely to binaural processing.

Lastly, the precedence effect test was used to assess the ability to fuse two sounds into a single auditory image^48^. In this test, two sounds of approximately equal loudness were presented to the two ears with a short interaural time delay. The fused sound image was perceived on the side of the leading sound and remains there until the delay became long enough for the listener to perceive two distinct sounds (an echo).

The aim of this study is to investigate the advantages of bilateral BC stimulation in terms of SRM and BILD among participants with symmetrical and asymmetrical sensorineural hearing loss. Additionally, the study will examine the participants’ ability to fuse sounds from both sides.

## Results

This study evaluated the threshold at which speech was understood 50% of the time in a noisy environment under three different conditions: when the speech and noise signals were both directly in front of the participant (S0N0), when the speech signal was in front while the noise was at a 45-degree angle to the side of the participant (S0N45), and when the speech signal was phase-inverted between the two ears while the noise signal was equal at both ears (S180N0). The study also measured the precedence effect, which was the perceived location of a sound source when the time delay between the sound from the two sides varied from 0 to 50 ms.

The study included 21 participants with symmetric hearing loss and nine participants with asymmetric hearing loss. Data from subjects with normal hearing, obtained from a study by Zeitooni et al.^28^, were included for comparison. All tests were conducted using both BC stimulation and AC stimulation through earphones.

**Fig. 1:**
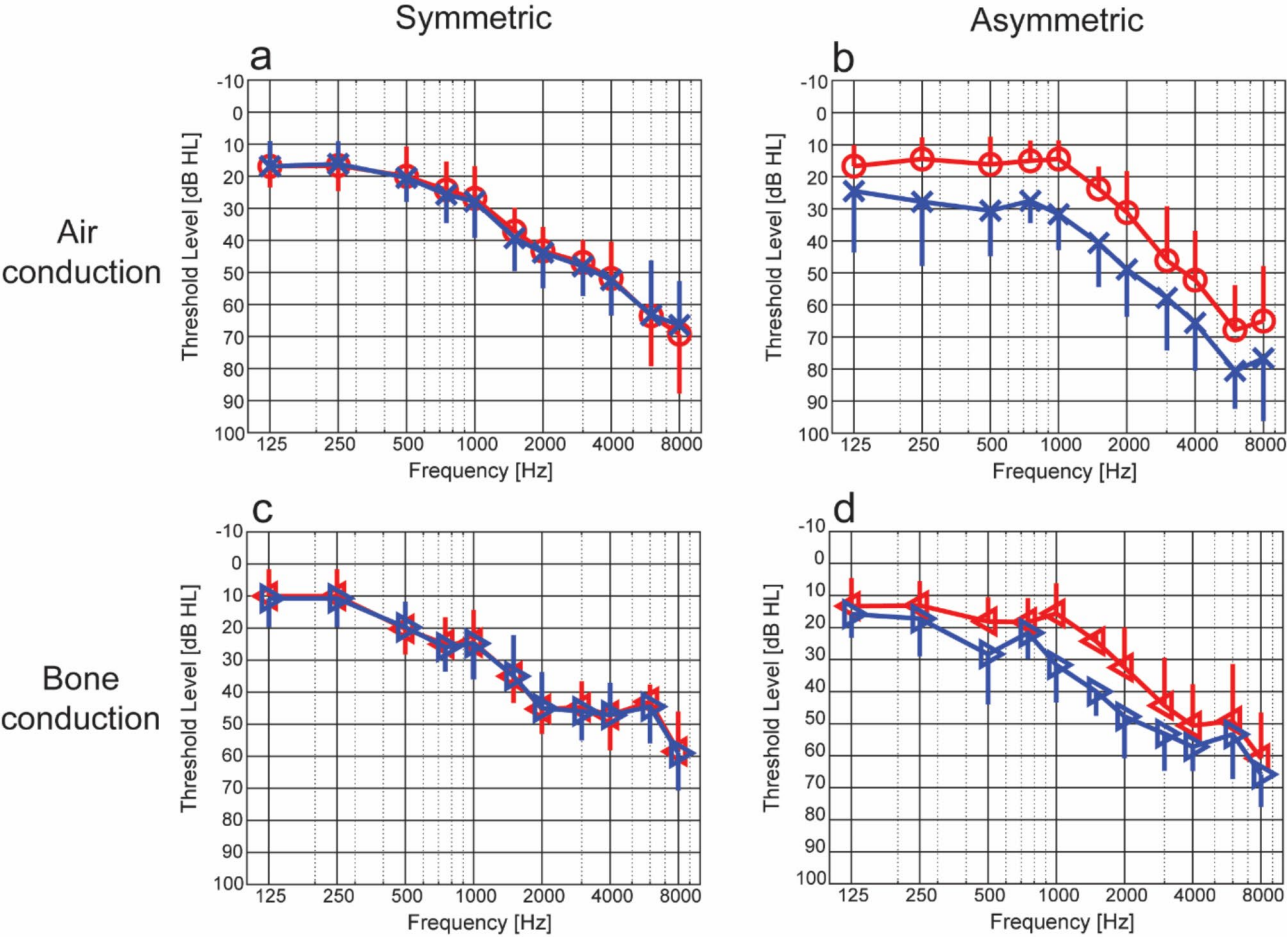
Average hearing thresholds for the two participant groups. Panels **(a)** and **(c)** present the symmetric group with AC and BC stimulation, respectively. Panels **(b)** and **(d)** present the asymmetric group with AC and BC stimulation, respectively. In the symmetric group, the right ear is represented by red lines and markers (circles and left-pointing triangles), while the left ear is represented by blue lines and markers (crosses and right-pointing triangles). In the asymmetric group, the better ear is represented by red lines and markers, and the worse ear is represented by blue lines and markers. The vertical lines denote 1 standard deviation.

Figure 1 shows the audiograms for all participants. In the asymmetric group, the better ear is designated as the right ear, and the worse ear as the left ear. The average AC PTA4 (0.5, 1, 2, 4 kHz) for the right ear in the symmetric group was 35.5 dB HL and for the left ear was 36.1 dB HL (Fig. 1a). With BC stimulation, the average right ear PTA4 for this group was 34.3 dB HL and the left ear was 34.2 dB HL (Fig. 1c). In the asymmetric group, the better ear had an average AC PTA4 of 28.2 dB HL and the worse ear a PTA4 of 45.6 dB HL (Fig. 1b). With BC stimulation, the average PTA4 was 29.2 dB HL in the better ear and 40.1 dB HL in the worse ear (Fig. 1d).

**Fig. 2:**
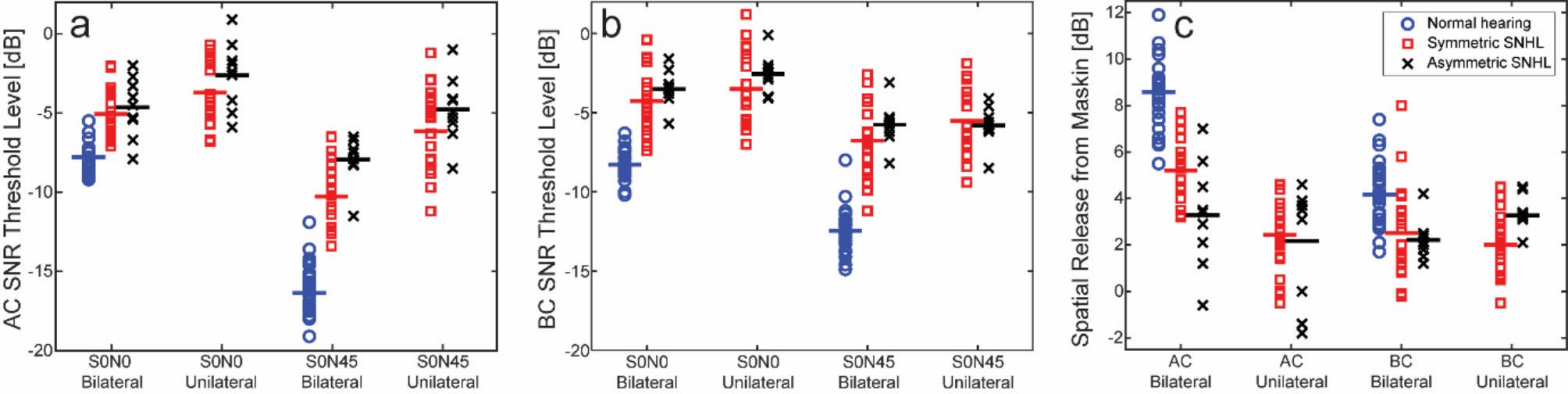
SNR thresholds at 50% correct word recognition for **(a)** AC stimulation and **(b)** BC stimulation. **(c)** displays the spatial release from masking (SRM). Participants with normal hearing are represented by blue circles, those with symmetric hearing loss by red squares, and those with asymmetric hearing loss by black crosses. The horizontal lines indicate the mean values.

The results of the speech-in-noise test, both with speech and noise co-located (S0N0) and with speech at the front and noise positioned at 45 degrees (S0N45), are shown in Fig. 2. Figure 2a displays the outcomes with AC stimulation, while Fig. 2b shows the results with BC stimulation. Individual data points are displayed, with blue circles representing participants with normal hearing (data from Zeitooni et al.^28^), red squares representing participants with symmetric hearing loss, and black crosses representing participants with asymmetric hearing loss. Averages are indicated by horizontal lines in Fig. 2, and the mean values along with standard deviations of all conditions are provided in Table 1. SNR thresholds are shown for both bilateral and unilateral stimulation in the two hearing loss groups. In the unilateral condition, stimulation was applied on the side with the more favorable SNR in the S0N45 condition, and to the better ear for participants with asymmetric hearing loss.

The general results presented in Fig. 2a-b indicate better outcomes for the normal hearing group compared to the hearing loss groups, followed by the symmetric hearing loss group, with the worst outcomes observed in the asymmetric hearing loss group. In general, bilateral stimulation yielded better results than unilateral stimulation, sound and noise separation (S0N45) led to better outcomes than collocated sound and noise (S0N0), and AC stimulation performed better than BC stimulation. These trends were analyzed using mixed-model ANOVAs.

Since the normal hearing group was not tested unilaterally, it was not possible to include all conditions in a single ANOVA. Therefore, the first ANOVA assessed the *Group* (normal, symmetric hearing loss, asymmetric hearing loss) as between-subjects factor, and *Mode* (AC, BC) and *Spatial* configuration (S0N0, S0N45) as within-subjects factors. All main effects were significant: *Group* [F(2,54) = 116.977, *p* < .001, η^2^ = 0.812], *Mode* [F(1,54) = 77.955, *p* < .001, η^2^ = 0.591], and *Spatial* [F(1,54) = 572.182, *p* < .001, η^2^ = 0.914]. Significant interactions were found for *Group* x *Spatial* [F(2,54) = 40.627, *p* < .001, η^2^ = 0.601], *Mode* x *Spatial* [F(1,54) = 85.655, *p* < .001, η^2^ = 0.613], *Mode* x *Spatial* x *Group* [F(2,54) = 16.936, *p* < .001, η^2^ = 0.288], while the interaction between *Mode* x *Group* was not significant (*p* = .489).

Posthoc analysis (Sidak) revealed that the normal hearing group had significantly better scores than both hearing-impaired groups (*p* < .001), though the two hearing-impaired groups did not significantly differ from each other (*p* = .078). The normal hearing group showed significantly better results with both AC and BC stimulation (*p* < .001). The symmetric hearing loss group approached significantly better outcomes compared to the asymmetric hearing loss group with AC stimulation (*p* = .051), but no significant difference was observed with BC stimulation (*p* = .35). Across all groups, results were significantly better with AC stimulation compared to BC stimulation (*p* < .001), and across both spatial configurations (S0N0, *p* = .014; S0N45, *p* < .001). No significant difference was found between the two hearing-impaired groups for the S0N0 condition (*p* = .55), but the symmetric hearing loss group performed significantly better than the asymmetric hearing loss group in the S0N45 condition (*p* = .025). All other pairwise comparisons between *Group* and *Spatial* configuration were significant (*p* < .001). Overall, the S0N45 condition resulted in better outcomes than the S0N0 for both AC and BC stimulation (*p* < .001).

These findings were primarily driven by the superior performance of the normal hearing group compared to the hearing-impaired groups, the better performance of AC stimulation compared to BC stimulation, and the improved outcomes with spatially separated sound and noise (S0N45) compared to collocated sound and noise (S0N0).

**Table 1:** The table presents the averages and standard deviations (in parenthesis) of the tests conducted on two groups with hearing losses, for both AC and BC stimulation. Data for individuals with normal hearing are derived from Zeitooni et al.^28^.

	Symmetric				Asymmetric				Normal	
	AC		BC		AC		BC		AC	BC
**Test**	Bilateral	Monaural	Bilateral	Monaural	Bilateral	Monaural	Bilateral	Monaural	Bilateral	Bilateral
**S0N0** [dB]	-5.1 (1.5)	-3.7 (1.9)	-4.3 (1.8)	-3.5 (2.2)	-4.6 (1.8)	-2,6 (2.0)	-3.5 (1.3)	-2.6 (1.4)	-8.0 (1.4)	-8.5 (1.2)
**S0N45** [dB]	-10.3 (1.9)	-6.2 (2.5)	-6.8 (2.5)	-5.5 (2.0)	-7.9 (1.4)	-4.8 (2.0)	-5.8 (1.4)	-5.8 (1.4)	-16.4 (1.8)	-12.5 (1.3)
**S180N0** [dB]	-10.2 (2.4)		-9.1 (2.9)		-7.8 (2.5)		-6.1 (2.2)		-14.7 (2.3)	-13.3 (2.6)
**SRM** [dB]	5.2 (1.3)	2.4 (1.5)	2.5 (1.9)	2.0 (1.2)	3.3 (2.2)	2.2 (2.4)	2.2 (1.0)	3.3 (1.0)	8.4 (1.5)	4.0 (1.5)

The second ANOVA included only the two hearing-impaired groups as the between-subjects factor (*Group*), with *Stimulation* (bilateral, unilateral) added as an additional within-subjects factor. Since the other factors (*Mode* and *Spatial* configuration) were analyzed in the previous ANOVA, this analysis focused on the *Stimulation* factor. It should be noted, when the normal hearing group was excluded from this analysis, the main factor of *Group* was no longer significant (*p* = .137), consistent with the previous findings. However, all other main effects remained significant (*p* < .001).

Pairwise comparisons between bilateral and unilateral stimulation revealed significant differences across most conditions, with p-values ranging from 0.001 to 0.006. The only exception was the S0N45 condition with BC stimulation in the asymmetric hearing loss group, where no significant difference was found between bilateral and unilateral stimulation (*p* = .959).

A separate analysis was conducted on the SRM, as shown in Fig. 2c. On average, SRM was approximately twice as high in dB for AC compared to BC stimulation: 8.4 dB versus 4.0 dB for the normal hearing group, 5.2 dB versus 2.5 dB for the symmetric hearing loss group, and 3.3 dB versus 2.2 dB for the asymmetric hearing loss group (Table 1). Bilateral stimulation generally resulted in better SRM compared to unilateral stimulation, except in the asymmetric hearing loss group with BC stimulation, where bilateral application led to worse results than unilateral application. Overall, the normal hearing group showed the best SRM while the symmetric hearing loss group had a slightly better SRM than the asymmetric hearing loss group.

The SRM was further analyzed using two mixed-model ANOVAs. The first analysis included all three groups, with the between-subject factor *Group* (normal hearing, symmetric hearing loss, and asymmetric hearing loss), and *Mode* (AC, BC) as within-subjects factor. This ANOVA revealed significant main effects of *Group* [F(2,54) = 40.687, *p* < .001, η^2^ = 0.601] and *Mode* [F(1,54) = 86.401, *p* < .001, η^2^ = 0.615], as well as a significant interaction *Mode* x *Group* [F(2,54) = 10.859, *p* < .001, η^2^ = 0.287]. Post-hoc (Sidak) analysis indicated that the normal hearing group differed significantly from both hearing-impaired groups (*p* < .001), while the two hearing impaired groups did not significantly differ from each other (*p* = .083). The difference between AC and BC stimulation was significant for the normal hearing and symmetric hearing loss groups (*p* < .001), but not for the asymmetric hearing loss group (*p* = .113).

The second ANOVA for SRM focused on the two hearing loss groups, with *Group* (symmetric, asymmetric) as between-subjects factor, and *Mode* (AC, BC) and *Stimulation* (bilateral, unilateral) as within-subjects factors. When the normal hearing group was excluded, the main effect of *Group* was no longer significant (*p* = .44), while *Mode* [F(1,28) = 6.338, *p* = .018, η^2^ = 0.185] and *Stimulation* [F(1,28) = 6.778, *p* = .015, η^2^ = 0.195] remained significant. Significant interactions were observed for *Mode* x *Group* [F(1,28) = 6.615, *p* = .016, η^2^ = 0.191], *Stimulation* x *Group* [F(1,28) = 6.161, *p* = .019, η^2^ = 0.180], and *Mode* x *Stimulation* [F(1,28) = 18.524, *p* < .001, η^2^ = 0.398], but the three-way interaction *Mode* x *Stimulation* x *Group* was not significant (*p* = .92). Pairwise comparisons for bilateral versus unilateral stimulation revealed that bilateral stimulation provided significantly better SRM outcomes only for the symmetric hearing loss group with AC stimulation (*p* < .001). Other comparisons did not show significant differences (AC asymmetric, *p* = .22; BC symmetric, *p* = .15; BC asymmetric, *p* = .096).

**Fig. 3:**
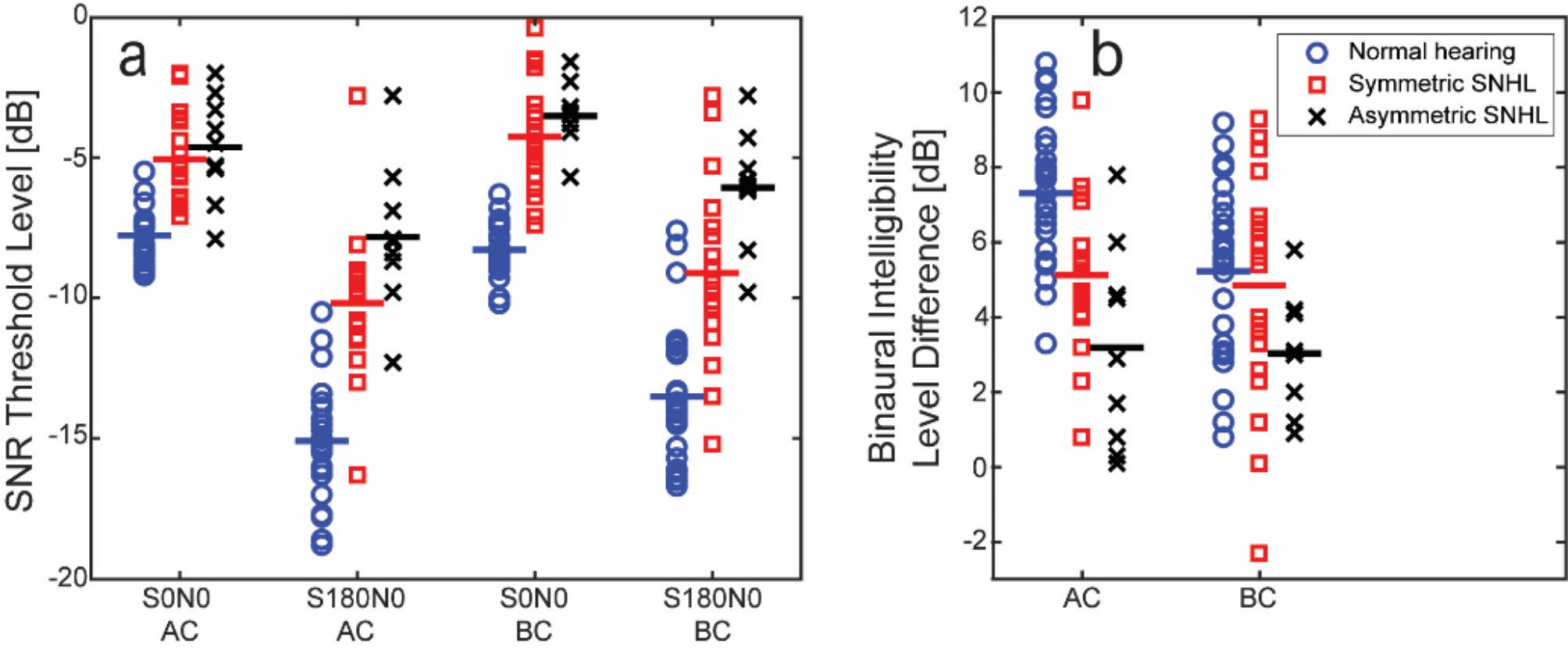
**(a)** SNR thresholds at 50% correct word recognition for co-located stimulation (S0N0) and for inverted speech between the ears (S180N0) with either AC or BC stimulation. **(b)** Binaural intelligibility level difference (BILD) for all participants with both AC and BC stimulation. Participants with normal hearing are represented by blue circles, those with symmetric hearing loss by red squares, and those with asymmetric hearing loss by black crosses. The horizontal lines indicate the mean values.

The BILD results are depicted in Fig. 3. Figure 3a presents the SNR thresholds for the S0N0 and S180N0 conditions with both AC and BC stimulation across all three groups. The S0N0 data are the same as those in Fig. 2. The general trend indicates improved SNR thresholds when the speech signal was presented out of phase between the ears (S180), better performance with AC compared to BC stimulation, and best results in the normal hearing group followed by the symmetric hearing loss group, with the asymmetric hearing loss group performing the worst.

Figure 3b shows the BILD results, indicating nearly identical outcomes for AC and BC stimulation in the two hearing loss groups. However, the symmetric group demonstrated approximately twice the binaural benefit compared to the asymmetric group (as detailed in Table 1). Even so, the normal hearing group exhibited a BILD that surpassed both hearing-impaired groups.

A mixed-model ANOVA was conducted on the BILD results, with *Group* (normal, symmetric, asymmetric) as between-subjects factor, and *Mode* (AC, BC) as within-subjects factor. This analysis revealed significant effect of *Group* [F(2,54) = 6.089, *p* = .004, η^2^ = 0.184], *Mode* [F(1,54) = 6.824, *p* = .012, η^2^ = 0.112], and a significant *Group* x *Mode* interaction [F(2,54) = 5.313, *p* = .008, η^2^ = 0.164]. Post-hoc analysis (Sidak) showed that the normal hearing group performed significantly better than the asymmetric hearing loss group (*p* = .003), although there was no significant difference between the normal hearing group and the symmetric hearing loss group (*p* = .326), nor between the two hearing loss groups (*p* = .093). The difference between AC and BC stimulation was significant in the normal hearing group (*p* < .001) but not in the symmetric hearing loss group (*p* = .585) or the asymmetric hearing loss group (*p* = .840).

**Fig. 4:**
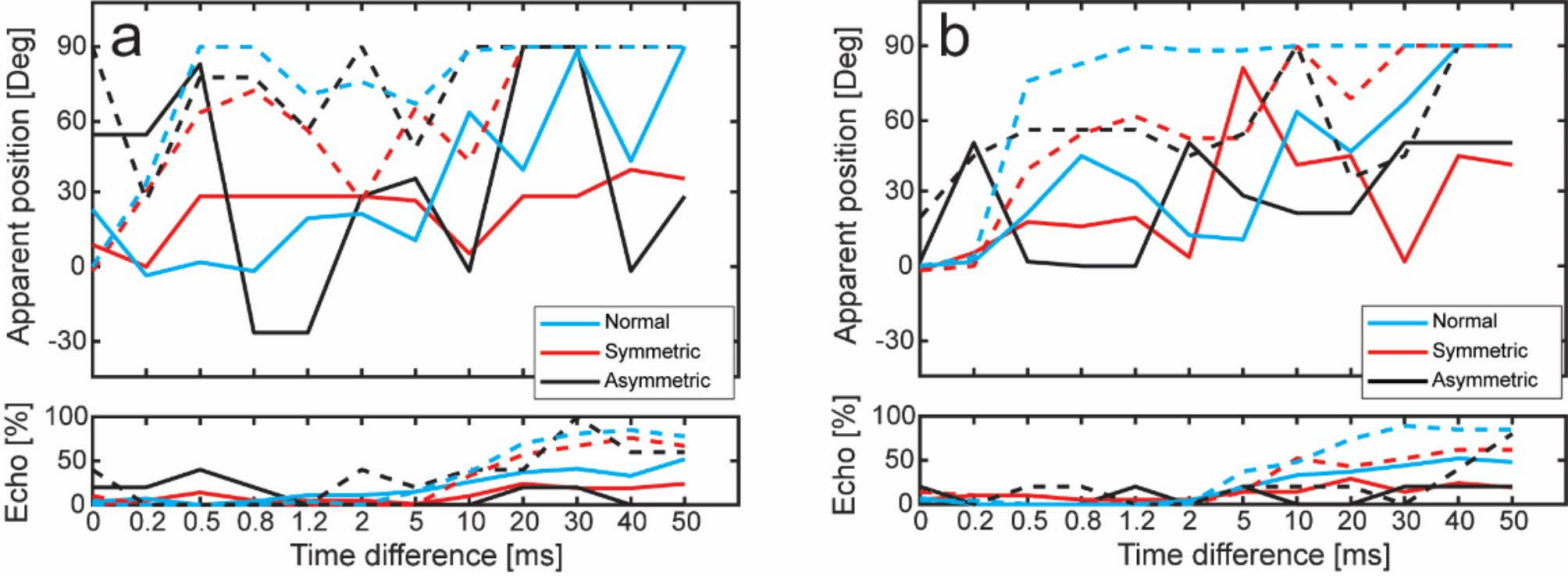
Median results of the precedence test with **(a)** low-frequency and **(b)** high-frequency stimulation. The upper panels depict the apparent position of the sound, while the lower panels show the percentage of participants perceiving an echo. Solid lines represent BC stimulation and dashed lines represent AC stimulation.

The outcomes from the precedence effect test are illustrated in Fig. 4a for low-frequency sound stimulation and in Fig. 4b for high-frequency sound stimulation. The upper panels depict the average perceived position of the sound as a function of time delay between the presentations at both ears, while the lower panels show the percentage of participants who perceived an echo of the sound (non-fused) as a function of time delay.

The results with AC stimulation in individuals with normal hearing (represented by the blue dashed line) followed the expected trend, moving from 0° at no time difference to full lateralization (90°) at a time difference of 0.5 to 0.8 ms, with echo perception starting at time difference of 10 to 20 ms. Both groups of participants with hearing loss followed a similar but less pronounced trend when stimulated by AC. Particularly, the high-frequency results in Fig. 4b show less lateralization effects compared to individuals with normal hearing. It is important to note that for the asymmetric group the stimulation at the worse ear was delayed, which should result in lateralization toward the better hearing ear. However, since the sound at the worse ear was amplified with a higher gain compared to the better ear, the higher gain in the worse ear could have affected the perception of sound lateralization.

When stimulated by BC, the perception of position with time delay was less distinct. In individuals with normal hearing (represented by the blue solid line), the lateralization effect began at time delays between 5 and 10 ms, reaching full lateralization at the highest time delays used (40 and 50 ms), with around half of them perceiving an echo. The two groups with participants with hearing loss did not show a clear pattern. With low-frequency stimulation in Fig. 4a, the median perception in the symmetric group increased to around 30° at 0.5 ms time difference and remained around 30° with increased interaural time differences. The asymmetric group did not show any specific trend with low-frequency stimulation and even reported perception of lateralization toward the delayed side (Fig. 4a). With high-frequency stimulation (Fig. 4b), both hearing loss groups showed more of a trend of increased lateralization with time delay, even though patterns vary. The median position perception was around 45° at the largest delays, and only a minority of participants reported perceiving an echo.

## Discussion

The present study explored the advantages of using bilateral BC sound in individuals with mild to moderate sensorineural hearing loss, which could be either symmetric or asymmetric. The hearing loss range examined in this study is common for BC hearing aid users^38,49^.

In most scenarios, our participants demonstrated improved speech intelligibility with bilateral stimulation compared to unilateral stimulation (Fig. 2). However, in the asymmetric group using BC stimulation, similar thresholds were achieved with both bilateral and unilateral stimulation in the S0N45 condition. This was because the noise was directed at the worse ear for the asymmetric participants, with stimulation at the better ear and a favorable SNR in the unilateral condition. Since the beneficial SNR was at the better ear, adding a worse ear with a poorer SNR was not expected to enhance speech intelligibility. However, if the noise was directed towards the better ear, bilateral stimulation would likely be beneficial. This was not tested in the current study.

In the symmetric hearing loss group, the BC SRM was similar for both unilateral and bilateral stimulation (Fig. 2c). The SRM in the unilateral condition was also similar for AC and BC stimulation in the symmetric group, as well as with AC stimulation in the asymmetric group. The only exception was the better SRM with unilateral BC stimulation in the asymmetric group. However, these differences were not statistically significant, suggesting that the improved speech perception in both the unilateral and bilateral conditions in the asymmetric group, and with BC stimulation in the symmetric group, could be attributed solely to the head shadow of the noise, or the better-ear effect. This finding was supported by a recent study where the speech stimulation was at one ear and the noise position varied between co-located and at the opposite ear^29^. In that study, when the noise position was at the contralateral side, the head shadow dominated the mechanism for spatial benefit.

The current study aligned with the findings in Zeitooni et al.^28^, which reported a better ear effect in unilaterally deaf participants. In the Zeitooni et al. study, the SRM with AC stimulation in unilaterally deaf participants was 2.1 dB, while it was 2.4 dB and 2.2 dB in the symmetric and asymmetric groups in the current study, respectively. The average SRM for the unilaterally deaf participants with BC stimulation in the Zeitooni study was 1.7 dB, while the current study showed unilateral SRM with BC stimulation of 2.0 dB and 3.3 dB in the symmetric and asymmetric groups. Both studies used the same test setup and speech material, but the Zeitooni et al. study provided the BC stimulation at the mastoid, while the current study gave the stimulation at a position further back on the skull where a typical implant for BC hearing aids is placed.

The current study demonstrated a significant benefit of using bilateral BC stimulation compared to unilateral stimulation. The improvement with bilateral stimulation using AC modality was approximately twice as large (in dB) compared to using BC stimulation (Fig. 2). This is consistent with previous studies using participants with normal hearing^27,28^. There was also a clear decrease in binaural benefit with increasing hearing loss, where the two groups including participants with hearing loss performed worse compared to results from participants with normal hearing, independent of stimulation modality. The reduction of binaural benefit with peripheral hearing loss with AC stimulation has been communicated previously^11^. However, a systematic investigation of the effect of peripheral hearing loss on binaural benefit with bilateral BC stimulation has not been presented. Even so, it was expected that the binaural benefit would be reduced because of peripheral hearing loss when stimulation was by BC, which was the outcome in the present study.

A noteworthy finding was that the asymmetric hearing loss group benefitted from bilateral application with AC stimulation but not with BC stimulation (Fig. 2c). Even though bilateral BC SRM averaged 2.2 dB, it was higher in the BC unilateral SRM condition (mean 3.3 dB). One issue with the asymmetric participants was that the different sides were amplified differently. The current study adopted a linear amplification rationale based on the hearing thresholds^50^. This was less of a problem for AC stimulation, where the interaural separation was large enough to prevent the amplified sound to affect the contralateral ear^51^. With BC stimulation, the interaural separation is typically between 0 and 10 dB^37,52,53^. This means that some of the BC sound at the better ear was dominated by the amplified sound provided at the contralateral ear. Additionally, research suggests that AC and BC sounds have different loudness growth functions, which can affect perceived sound levels at each ear when using BC stimulation^54,55^. Consequently, how to set amplification for bilaterally fitted BC hearing aids in individuals with asymmetric hearing loss was not clarified and would be a topic for its own investigation.

The current study examined the effect of inverting the phase of the speech signal between the ears (BILD), and found that the benefit was largely independent of the stimulation modality (Fig. 3b). In the BILD testing, there was no better-ear effect since only the phase information of the speech signal was manipulated. The benefit was more pronounced in the symmetric hearing loss group compared to the asymmetric hearing loss group, but was significant for both groups and stimulation modalities. Interestingly, the benefit of inverting the speech signal with BC stimulation in the symmetric group was similar to the result with inverting the speech signal with BC stimulation in the group of normal hearing participants in the study by Zeitooni et al.^28^, shown in Fig. 3. However, the normal hearing participants reported better BILD results with AC stimulation compared to the participants with symmetric hearing loss. It should be noted that Zeitooni et al.^28^ reported the benefit of the BILD test in unilateral deaf individuals to be 2.4 dB with BC stimulation. Therefore, even if the SNR at the stimulation position was unchanged, inverting the speech signal could result in superposition of the bilateral BC stimulation at the cochlear level, enhancing the intelligibility^28^.

The precedence data in the current study aligned with previous results when stimulation was by BC^27,28^ (Fig. 4). With AC stimulation, the perception was fused and directed toward the leading stimulation (dashed lines in Fig. 4). The perception was close to fully lateralized after 0.5 to 0.8 ms lag-time for the group consisting of normal hearing individuals, while the groups of hearing-impaired individuals showed less consistent lateralization. When the stimulation was by BC, the lateralization did not appear until a delay of 10 ms for individuals with normal hearing and no systematic lateralization for the groups with hearing-impaired people. One possible reason for this lack of distinct lateralization with BC stimulation was the cross-head transmission with BC stimulation.

A recent study estimated the interaural time-delay of BC cross-head transmission to be approximately 0.06 ms at 250 Hz, increasing to 1.1 ms at 0.8 kHz, and decreasing to 0.6 ms at 4 kHz^42^. Given that the transcranial attenuation was primarily less than 10 dB within this frequency range, the impact from cross-head transmission was substantial. This could lead to confusion when trying to identify the position of a sound when stimulation was by BC. As a result, a significant proportion of individuals listening through bilateral BC may encounter difficulties localizing a sound source.

While the current study suggests that individuals listening through bilateral BC may have difficulties localizing a sound source, previous studies have shown that individuals with bilaterally applied BC sound can exhibit localization abilities^31,35,56^. This discrepancy may be partly due to adaptation. In the current study, participants were acutely fitted with BC sound without any adjustment period for this stimulation modality. However, individuals who have been fitted with BC hearing aids may, over time, develop better abilities to use binaural information, even if it has been distorted by cross-head BC sound transmission.

Other factors may also contribute to these differences. For instance, many of the studies using BC hearing aids in clinical patients involved patients with predominantly conductive hearing loss^31,35,57–59^. These patients typically have minor sensorineural hearing loss, and their BC thresholds were often better than 20 dB HL. As a result, these patients were more similar to the normal hearing participants than the participants with hearing loss in the current study. Nevertheless, the BC results of lateralization and localization in individuals with normal hearing were inferior to those with AC stimulation in participants with mild to moderate hearing loss.

Another possible discrepancy between studies on localization ability with bilateral BC stimulation and the data in the current study was the use of artificial conductive hearing loss. This was typically achieved by occluding the ear canals, often with earplugs^33,60,61^. However, this could lead to an enhancement of the ear canal sound pressure due to the occlusion effect^62^. As a result, ear canal sound pressure enhancement may have led to a greater separation of the ears, and the stimulation became more akin to AC than BC at low frequencies when the ear canal pathway was enhanced compared to other pathways for BC hearing^63^. A similar enhancement of the ear canal sound pressure leading to greater interaural separation was found when the BC transducer was placed close to the ear canal opening^64^. Additionally, ear surgery could also increase the interaural separation, resulting in improved binaural ability^65^.

A limitation of the present study was the small sample size of participants with asymmetric hearing loss. This restricted the applicability of the findings, and some of the statistically insignificant results for this group may have been attributed to the limited participant numbers rather than an absence of binaural effects. The stringent inclusion criteria, which specified acceptable hearing thresholds and interaural differences, made it challenging to identify suitable participants from the database. Regrettably, the constraints of the BC transducers meant that larger threshold ranges could lead to distorted stimulation due to amplification, or inaudibility if less compensation for the hearing loss was applied. To ensure both audibility and undistorted stimulation, the restrictive inclusion criteria were necessary. Despite these limitations, the group with asymmetric hearing loss demonstrated a significant spatial benefit, although this benefit was not superior in bilateral compared to unilateral stimulation in the current setup.

Clinically, this study demonstrated that bilateral BC sound application was superior to unilateral application, regardless of whether the benefit resulted from better-ear listening or binaural integration (Fig. 2). The findings indicated that the bilateral advantage of BC sound was evident in participants with both symmetric and asymmetric sensorineural hearing loss. As a result, the study suggests that patients eligible for BC hearing aids would perform better with two hearing aids rather than just one, especially if the symmetric or asymmetric sensorineural component was no worse than a PTA4 of 35 dB HL.

## Conclusion

The study demonstrated that individuals with mild to moderate hearing loss derived benefits from the bilateral application of BC sound compared to unilateral application. However, this binaural benefit was less pronounced compared to individuals with normal sensorineural hearing, and a symmetric hearing loss yielded a larger benefit than an asymmetric hearing loss. Moreover, even though the binaural benefit with bilateral BC stimulation was significant, it was approximately half (in dB) compared to the same benefit when stimulation was by AC. A large part of this benefit was attributed to the reduction of noise from the head shadow at the ear with a favorable SNR. However, binaural processes that improve speech intelligibility were also present, as evidenced by the enhanced perception in the BILD test. The ability to lateralize a sound to the leading side with interaural time delays was worse with BC stimulation compared to AC stimulation, and even worse when a hearing loss was present.

## Methods

The study was approved by the Regional Ethical Review Board in Linköping, Sweden (2012-168-32). All participants signed an informed consent prior to the testing and were made aware of their right to withdraw from the study at any point. All methods used follow clinical routine and the tests were conducted according to relevant guidelines and regulations.

Two groups of participants were recruited to the study. The first group comprised individuals with symmetrical mild to moderate sensorineural hearing loss. The inclusion criteria for this group were as follows: participants had to be 18 years or older, having a BC PTA4 (average of thresholds at 0.5, 1, 2, and 4 kHz) between 30 and 45 dB HL, an interaural BC PTA4 difference of no more than 6 dB, and a maximum PTA4 air-bone gap of 6 dB in both ears. Additionally, participants were required to be native Swedish speakers. Exclusion criteria included a history of ear surgery or being implanted with bone conduction hearing aids or their fixtures. Potential participants were identified from the clinical database of Linköping University Hospital, Linköping, Sweden. Of those invited, 21 individuals chose to participate. These participants ranged in age from 66 to 76 years, with an average age of 72 years, and included 13 females. Their average BC PTA4 was 34.3 dB HL (SD 3.0 dB) in the right ear and 34.2 dB HL (SD 3.5 dB) in the left ear.

The second group consisted of individuals with asymmetrical hearing loss. The inclusion criteria for this group were a BC PTA4 between 25 and 35 dB HL in the better ear, an interaural BC difference of 10 to 20 dB, and a maximum PTA4 air-bone gap of 6 dB in both ears. As in the first group, participants had to be native Swedish speakers and were excluded if they had a history of ear surgery or had been implanted with bone conduction hearing aids or their fixtures. Recruiting for this group proved more challenging, and nine individuals chose to participate. The participants in this group ranged in age from 43 to 74 years, with an average age of 68 years, and included one female. The average BC PTA4 thresholds were 29.2 dB HL (SD 4.5 dB) in the better ear and 40.1 dB HL (SD 4.8 dB) in the worse ear. In two participants, the worse ear was the right ear, while in seven, it was the left ear. Figure 1 presents the average audiograms and standard deviations for both ears in both groups where the better ear is coded as right ear for the asymmetric group.

For additional data, results from 27 individuals with normal hearing and otologically healthy ears, with no history of ear surgery, were included in the analysis. These individuals had participated in a previous study using the same test protocols as the current study^28^. Their results for the BCHA stimulation position in that study were used, as it is the same BC stimulation position as in the current study. These participants had hearing thresholds of 20 dB HL or better for AC (125 Hz to 8 kHz) and BC (250 Hz to 4 kHz), with air-bone gaps of 10 dB or less at all frequencies. The age range of the group was 22 to 44 years, with a mean age of 29.4 years, and 10 participants were female.

All tests were conducted in a sound-insulated test booth. Baseline AC and BC thresholds were measured using a Madsen Astera^2^ (Natus Medical ApS, Denmark), equipped with TDH-39 earphones and a Radioear B-71 BC transducer. Computer-based speech-in-noise tests and the precedence test were implemented in Matlab (MathWorks, US), with an external 24-bit soundcard (Asus Xonar Essence One) serving as interface. Testing with AC sound was conducted using a pair of Sennheiser HDA200 audiometric headphones (Sennheiser electronic GmbH, Germany) connected to the soundcard.

The BC transducers used were two Cordelle 2 (Cochlear BAS, Sweden) devices, attached to an elastic softband with two plastic circular interfaces of approximately 15 mm diameter. The interfaces were positioned approximately 55 mm behind the ear canal opening, in line with the upper part of the pinna, mirroring the placement of a percutaneous BC hearing aid. The static force exerted by the BC transducers from the soft band was approximately 3 Newtons.

**Fig. 5:**
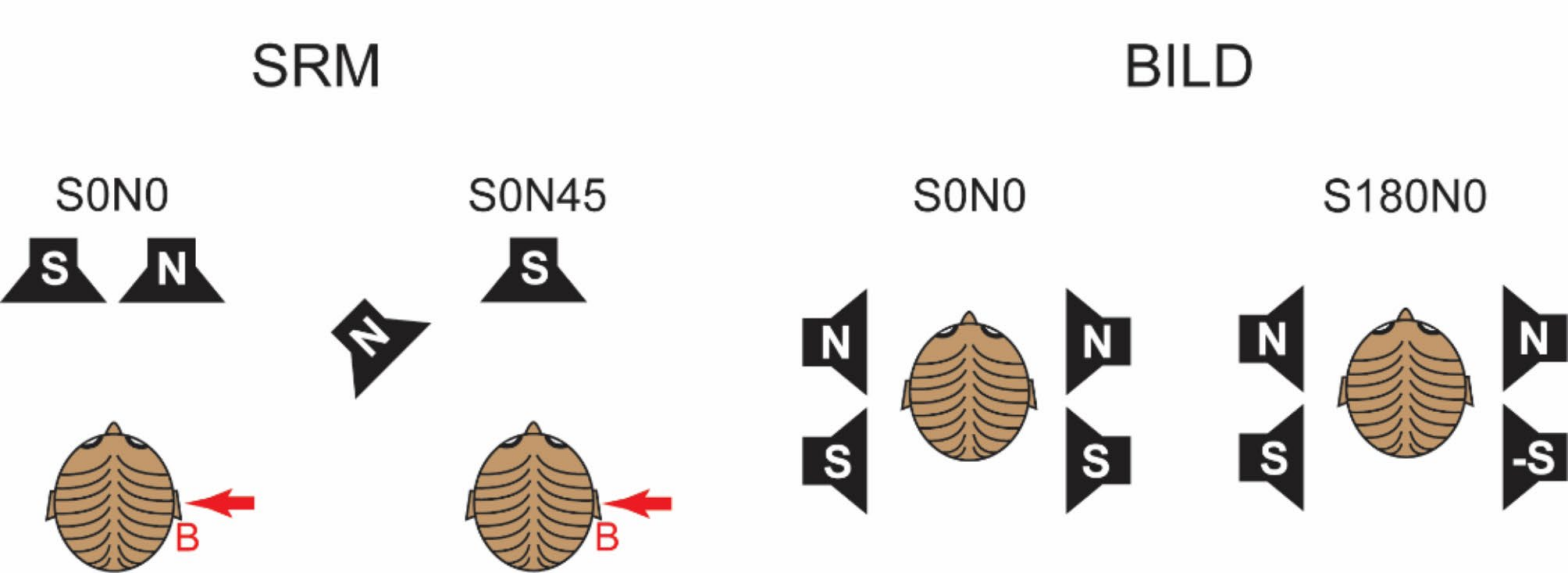
The simulated measurement configuration for SRM and BILD testing. All tests are done with AC and BC headsets, and generic HRTFs are used to filter the signals at each ear. The red B indicates the better ear for the asymmetric hearing loss participants and the red arrow indicates the side for unilateral stimulation.

The Swedish Hagerman’s sentences^66^, also known as the matrix test, was used for speech-in-noise testing. The speech was delivered by a female voice, and the noise was speech-weighted with 10% modulation. Each sentence contained five words that were semantically correct but had low predictability. Each list contained ten sentences, and two lists were used to test each condition. The aim of the test was to determine the SNR at which the participant could correctly repeat 50% of the words in the sentences, based on an adaptive algorithm^67^.

The speech-in-noise test was conducted under three conditions: S0N0, S0N45, and S180N0 (Fig. 5). In the S0N0 condition, the speech and noise were identical at both the left and right transducers, creating the perception that the sound originated from the front. In the S180N0 condition, the speech signal was phase-inverted between the two sides, while the noise remained identical on both sides. In the S0N45 condition, the speech signal was equal at both sides, also appearing to originate from the front, but the noise was spatially shifted 45 degrees to one side. This simulated spatial shift was achieved by filtering the noise differently at each side using a generic head-related transfer function model^68^. As a result, the noise signal differed between the ears in this condition. Figure 5 illustrates the simulated spatial configurations for each condition. The actual stimuli were delivered through AC or BC headsets, maintaining the spatial configuration based on head-related transfer function filtering.

The study examined SRM by comparing the SNR thresholds in two conditions: S0N0 and S0N45 (Fig. 5). These tests were conducted for two stimulation modalities (AC and BC) and two situations (bilateral and unilateral), resulting in each condition being tested four times (2 conditions x 2 situations). Binaural intelligibility level difference (BILD) was evaluated using SNR thresholds for S180N0 and S0N0. The S180N0 condition was tested only with bilateral sound application for both AC and BC stimulation. This meant that each participant underwent speech-in-noise tests ten times. To prevent the need for refitting the headsets, each participant completed all tests in one modality (either AC or BC) before switching to the other modality. Within each group, half of the participants began with AC testing, while the other half started with BC testing. To minimize potential order effects in the speech-in-noise tests, the order of conditions within each group was counterbalanced across participants as much as possible.

In the unilateral stimulation condition for S0N45, the signal was presented to the transducer or headphone on the side with the better SNR, which was opposite the noise source, as indicated by the red arrow in Fig. 5. During AC stimulation, the non-stimulated ear was blocked with an earplug to ensure that only one ear was engaged. For BC stimulation, the opposite ear was left open to avoid an occlusion effect^62^. In the group with symmetric hearing loss, noise in the S0N45 condition was directed toward the right ear, while the left ear had the better SNR. In the group with asymmetric hearing loss, noise in the S0N45 condition was directed towards the worse ear, with the signal containing the better SNR presented to the better ear (indicated by the red B in Fig. 5). Additionally, in unilateral testing, the sound was always delivered to the better ear.

The speech level was set at 60 dB SPL for the earphones, with the noise level adaptively altered during testing. This speech level corresponds approximately to 40 dB HL, and the same level was used for the BC testing. To ensure audibility and compensate for the participants’ hearing loss, the Cambridge formulae for linear amplification was used^50^. Each participant’s audiogram for the ears separately were used to amplify the delivered sound. Consequently, for the participants in the asymmetric group, the worse ear received higher amplification of the sound than the better ear. Moreover, the same amplification was used for sounds delivered by AC and BC. For details on how the AC and BC stimulation were equalized, see Zeitooni et al.^28^.

The study investigated the precedence effect using noise bursts of high and low frequencies, each lasting 1 s. The noise-bursts had 1 ms sine-squared rise and fall. The low-frequency noise ranged between 0.4 and 0.6 kHz, while the high-frequency noise ranged between 3.0 and 5.0 kHz. The experiment included twelve different interaural time delays, ranging from 0 to 50 ms. Participants were instructed to identify the perceived location of the sound, which could be on the left side (-90 degrees), in front (0 degrees), or on the right side (90 degrees). They indicated their perception on a computer screen by marking the location with a ruler, within a range of -90 to 90 degrees.

In the symmetric group, the delayed sound was on the left side, while in the asymmetric group, the delayed sound was at the worse ear. As a result, it was expected that the lateralization would be towards the right side for the symmetric group and toward the better ear in the asymmetric group. In the results, the better side was coded as the right side for the asymmetric group. If the sound was perceived as two separate sounds, participants were instructed to report this as an echo. The experiment involved four runs of the precedence effect test, with participants tested with low and high-frequency stimulation using both AC and BC sound.

The testing sequence was as follows: (1) pure-tone hearing threshold measurements, (2) ten speech-in-noise conditions, and (3) four precedence effect test conditions. The total testing duration for each participant ranged from 1.5 to 2 h.

The output of the transducers was measured and verified before the testing. The audiometer was calibrated according to ISO 389 − 1^69^ and ISO 389 − 3^70^ for measurement of hearing thresholds. The output from the headphones with the computer-based stimulation was measured on an ear simulator (IEC 60318-1^71^) and with BC stimulation on an artificial mastoid (Brüel & Kjær type 4930) connected to an analyzer (Brüel & Kjær type 2250). Based on these measurements, the outputs of the left and right transducers were corrected to deviate at most 1 dB at one-third octave frequencies between 125 Hz and 8 kHz.

The results were analyzed using mixed-model ANOVAs with Sidak post hoc corrections, computed with IBM SPSS Statistics (ver. 29).

## Data Availability

The data presented in this study are available on request from the corresponding author.
